# Comparing Walking-Related Everyday Life Tasks of Children with Gait Disorders in a Virtual Reality Setup With a Physical Setup: Cross-Sectional Noninferiority Study

**DOI:** 10.2196/49550

**Published:** 2024-03-18

**Authors:** Sophia Rhiel, Andrina Kläy, Urs Keller, Hubertus J A van Hedel, Corinne Ammann-Reiffer

**Affiliations:** 1 Swiss Children’s Rehab, University Children's Hospital Zurich University of Zurich Affoltern am Albis Switzerland; 2 Children’s Research Center, University Children’s Hospital Zurich University of Zurich Zurich Switzerland

**Keywords:** adolescent, child, gait, head-mounted display, motion capture, neurological rehabilitation, noninferiority trial, physical therapy, virtual reality, walking

## Abstract

**Background:**

A frequent rehabilitation goal for children with gait disorders is to practice daily-life walking activities. Unfortunately, these are often difficult to practice in a conventional therapeutic setting. Virtual reality (VR) with head-mounted displays (HMDs) could be a promising approach in neurorehabilitation to train such activities in a safe environment. First, however, we must know whether obstacles in VR are indeed mastered as obstacles.

**Objective:**

This study aimed to provide information on whether VR is feasible and motivating to induce and practice movements needed to master real obstacles in children and adolescents with gait disorders. Furthermore, this project aims to evaluate which kinds of everyday walking activities are appropriate to be practiced in VR.

**Methods:**

In this cross-sectional study, participants stepped over a bar, crossed a gap, balanced over a beam, and circumvented stationary obstructions arranged in a course under real physical and virtual conditions wearing a VR HMD. We recorded the respective primary outcomes (step height, step length, step width, and minimal shoulder-obstacle distance) with motion capture. We then calculated the mean differences and 95% CI of the spatiotemporal parameters between the VR and physical setup and later compared them using noninferiority analysis with margins defined a priori by a clinical expert panel. Additionally, the participants responded to a standardized questionnaire while the therapists observed and evaluated their movement performance.

**Results:**

We recruited 20 participants (mean age 12.0, range 6.6-17.8 years) with various diagnoses affecting their walking ability. At 3.77 (95% CI 1.28 to 6.26) cm, the mean difference in step height of the leading foot in the overstepping task did not exceed the predefined margin of –2 cm, thus signifying noninferiority of the VR condition compared to mastering the physical obstacles. The same was true for step length (–1.75, 95% CI –4.91 to 1.41 cm; margin –10 cm), step width (1.05, 95% CI 0.20 to –1.90 cm; margin 3 cm), and the minimal shoulder-obstacle distance (0.25, 95% CI –0.85 to 0.35 cm; margin –2 cm) in the other tasks. Only the trailing foot in the overstepping task yielded inconclusive results.

**Conclusions:**

Children with gait disorders perform everyday walking tasks like overstepping, crossing, balancing, or circumventing similarly in physical and VR environments, suggesting that VR could be a feasible therapeutic tool to practice everyday walking tasks.

## Introduction

In pediatric neurorehabilitation, children and adolescents with congenital or acquired lesions of the sensorimotor system often experience impairments in gait [1,2]. Consequently, recovery of walking ability is a frequent rehabilitation goal in pediatric neurorehabilitation [3]. Thereby, the focus is on promoting everyday life activities and ensuring meaningful participation for the child and their family [4]. Therapies targeting gait encompass a wide variety of therapeutic approaches. In our clinic, Swiss Children’s Rehab, these therapies include, for example, conventional physical therapy, including task- and everyday life–oriented training, rehabilitation robots, and sports therapy. Normally, these therapies occur in a conventional therapeutic setting. However, within this setting, many everyday walking tasks, such as, for example, crossing a wide gap to board public transportation or avoiding contact with people or obstacles while navigating through crowded places, cannot be reasonably practiced.

In recent years, immersive virtual reality (VR) has become increasingly popular. Since companies have made the technology more accessible to the community through more affordable and easy-to-use devices, the use of VR has increased, as have the areas of its use [5]. Accordingly, this upswing in VR could be promising for its implementation in neurorehabilitation. Immersive VR puts users directly into virtual scenarios and gives the illusion of a full physical presence, providing rich sensory fidelity (high degree of reliability) [6,7]. To experience immersive VR, head-mounted displays (HMDs) are most suitable and can convey many of the abovementioned impressions [8]. A potential goal of using VR in pediatric neurorehabilitation could be to enhance children’s abilities in their daily lives by practicing task-specific activities relevant to their everyday lives while still being in a safe therapeutic environment. Furthermore, its game-like attributes and animations can increase children’s motivation and enhance their active participation by minimizing their focus on task repetitions [9,10]. Additionally, as VR is an accessible and affordable technology, it could enable home training. Moreover, a significant advantage of using VR in children aged between 6 and 18 years could be that they experience higher levels of presence and “realness” within a virtual environment compared to adults [11].

Recent studies have already investigated the effectiveness of acquiring different cognitive and motor tasks with VR. In the pediatric field, VR has been mainly used for pain management [6] or educational purposes [12,13], as well as to create relaxing and learning opportunities for children diagnosed with autism spectrum disorder [14,15] or attention deficit hyperactivity disorder [16]. However, the long-term effects of VR on developing children are unknown, and cybersickness or fatigue of the eyes and brain are potential disadvantages [6,17,18]. According to the authors’ best knowledge, no evidence exists of using immersive VR as a gait therapy intervention in children with gait disorders. When including results from augmented reality studies, a systematic review showed moderate evidence for improved gait-related outcomes when gait training was enhanced with commercially available videogame systems, such as the Nintendo Wii or Microsoft Xbox Kinect, in children with cerebral palsy (CP) [19]. Furthermore, a systematic review and meta-analysis from Chen et al [20] showed a large effect size of *d*=0.861 for improved motor function in children with CP when comparing commercially available game systems with conventional therapy or controls (eg, no intervention). However, such systems lack essential aspects of VR since they are usually presented on a 2D screen or as floor projections [8] and, therefore, do not transmit the entire concept of VR, including full physical presence and immersion.

Immersive VR offers many advantages regarding task-specific training, motivation, “realness,” and costs [5-7]. Still, it remains uncertain whether the use of VR in children with gait disorders is a feasible approach to inducing and practicing the movements required to perform everyday gait activities. Reasons to assume that VR in children with gait disorders might not be feasible are the lack of visual information of the lower extremities and the difference in the perception of virtual obstacles by the children [6,11,21]. Therefore, a prerequisite for the meaningful use of VR in training everyday gait activities would be that the children master obstacles presented in VR like they master physical obstacles. Thus, this project aims to provide information on whether a VR setup is feasible and motivating to induce and practice movements that are needed to master real obstacles in children and adolescents with gait disorders. Furthermore, this project aims to evaluate which kinds of everyday walking activities are appropriate to be practiced in such a VR setup. To evaluate this, we compare the spatiotemporal parameters of performing certain everyday walking tasks in a virtual and a physical environment using a noninferiority analysis. The noninferiority analysis should indicate that the virtual setup is not unacceptably worse than the physical setup.

## Methods

### Ethical Considerations

This cross-sectional study took place at the gait laboratory of Swiss Children’s Rehab, University Children’s Hospital Zurich, during a single 60-minute session. The ethics committee of the Canton of Zurich confirmed through a clarification of responsibility that no approval was needed for this study (Req-2021-00364).

### Participants

We included children and adolescents aged between 6 and 18 years with gait disorders undergoing inpatient or outpatient rehabilitation at Swiss Children’s Rehab. In line with recommendations for comparative studies, which propose 8 to 25 participants [22], we aimed to include 20 participants. All children who were receiving physiotherapy at the time of recruitment were screened according to the inclusion and exclusion criteria and recruited consecutively within 3 months. To be eligible to participate, they had to be able to walk short indoor distances without assistive devices or with crutches. Additionally, they had to be able to follow simple verbal instructions. Exclusion criteria were a history of seizures, epilepsy, blindness, or inability to use the HMD (eg, cybersickness, open wounds on the head).

Participants’ characteristics were collected from the patient records. The physiotherapist rated the functional mobility level using 2 performance measures: the Functional Mobility Scale (FMS) and the Gillette Functional Assessment Questionnaire (FAQ) walking scale [23]. The FMS describes the participant’s level of functional mobility by assessing the assistive device used in everyday life over 5 m, 50 m, and 500 m on a scale from 1 (uses a wheelchair) to 6 (independent on any terrain). The FAQ assesses functional walking abilities on a scale from 1 (can not make any steps at all) to 10 (walks, runs, and climbs on even and uneven terrain). Finally, the lower extremity proprioceptive impairments of the participants were rated with the percentage score of the proprioception subsection of the Fugl-Meyer (FM) assessment for the lower extremities [24]. We assessed proprioception at the hip, knee, ankle, and toe joints while the participant was supine and barefoot.

According to good clinical practice standards, we obtained written informed consent from the participants and their legal representatives before participation.

### Experimental Setup

**Figure 1:**
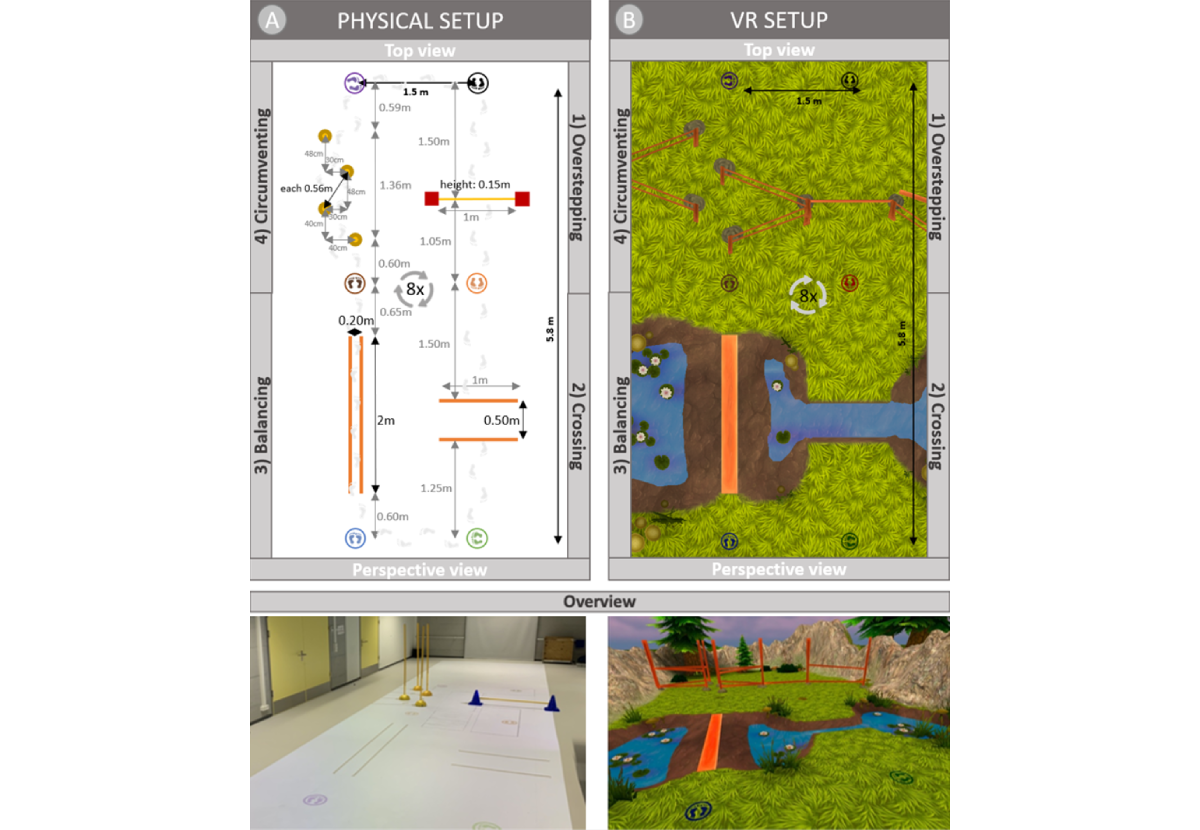
(A) Scheme of physical setup, and (B) the appearance of the VR setup when wearing the HMD.

The participants had to perform everyday walking tasks in 2 different conditions: physical setup and VR setup. In the physical setup, the participants had to master real (physical) obstacles (Figure 1A). The 4 obstacles, including overstepping, crossing, balancing, and circumventing, were arranged in a course. In the VR setup, the participants had to master the same 4 obstacles virtually. The obstacles were incorporated into an everyday environment (Figure 1B). The VR setup matched the locations and dimensions, but not the appearance of the physical obstacles. This discrepancy was chosen intentionally since we wanted to incorporate the obstacles into an everyday environment as they would appear in future applications. During the development process, it was ensured that the environment was designed as stimulatingly as possible, since interaction and sensorimotor contingencies are crucial contributors to a full VR experience [8]. Nevertheless, to compare the 2 conditions, we also had to keep the VR environment simple to avoid the participants being distracted from their tasks.

For this setup, the commercially available VR HMD Meta Quest 2 (Meta Platforms) was used. We aligned the coordinate systems of the physical and the virtual world, using the hand-tracking function of the Meta Quest, and scaled and rotated the virtual world based on 2 points. To test the alignment between the 2 conditions, we checked that the scaling coefficient was near 1.0.

To minimize the influence of fatigue, we randomized the sequence of the conditions and the starting position within the obstacle course. We used a minimization method (randomization factor 1), including the factors of gender, age, and functional walking ability defined by the FAQ. During the session, the physiotherapist accompanied the participants to ensure their safety and provide assistance if necessary.

### Task Description

**Figure 2:**
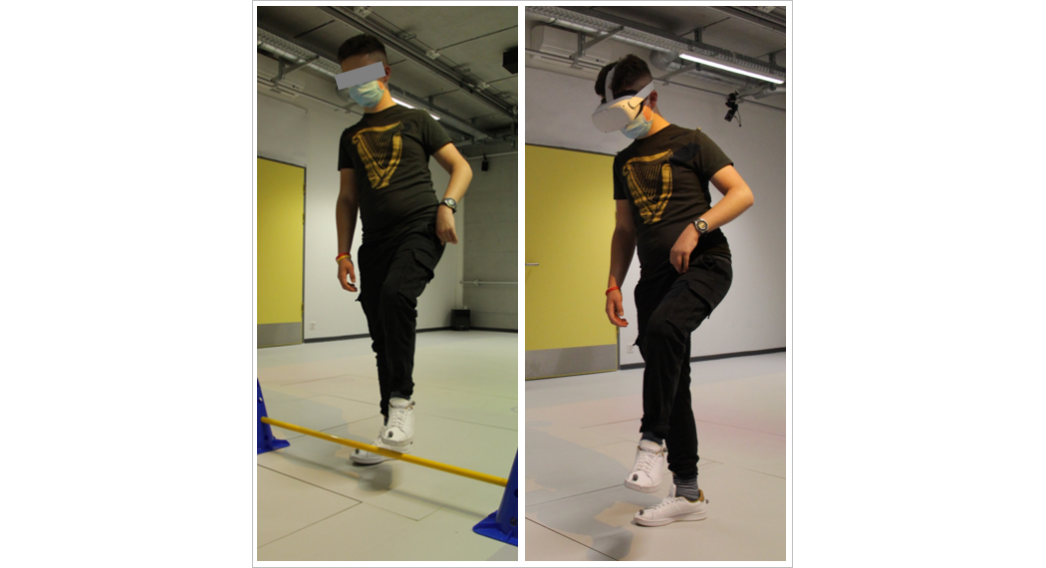
Execution of the overstepping tasks in the physical and virtual reality setups.

For the overstepping task, the participants had to step over a 15-cm-high obstacle, which consisted of a plastic bar mounted on 2 cones (physical setup) or the lower part of a fence (VR setup; Figure 2). In the physical setup, participants had to cross two 3-cm-wide lines projected on the ground with a beamer, whereas they had to cross a small stream in the VR setup. In both setups, the gap was 50 cm, thus exceeding the average step length of children with CP aged between 7 and 14 years (Gross Motor Function Classification System [GMFCS] levels I and II) or traumatic brain injury (TBI) [25-27]. For the balancing task, we instructed the participants to walk between two 2-cm-wide lines projected 20 cm apart on the floor in the physical setup and a 20-cm-wide wooden panel over a pond in the VR setup. Circumventing was performed by walking around 4 plastic poles (physical setup) or fence posts (VR setup). The distance of the poles was 56 cm, corresponding to approximately 1.7 times the average shoulder width of children aged between 6 and 18 years [28,29]. With an estimated protective zone of 30 cm around the obstacle [30], even smaller participants would sidestep, while taller participants could still pass through the obstacles, even when relying on crutches. In addition to the 4 tasks, the participants walked 6.5 m in a straight line without any obstacles, both with the HMD (walking on green grass) and without the HMD.

### Measurement Procedure

Task execution was recorded with a total of 12 Vicon Vero 2.2 high-speed cameras (Vicon Motion Systems). We placed 9 infrared reflective markers of 16 mm diameter on specific anatomical landmarks at both feet (3 markers each) and shoulders (3 markers). The markers were attached to the shoes as the participants performed the tasks with shoes and orthotics (if needed) as in everyday life.

After measuring the participants’ height and shoulder width and attaching the 9 reflective markers to the defined positions, the measurements started with either the physical or the VR condition. The participants first walked 4 times along the 6.5-meter walkway at self-selected walking speeds. Afterward, they performed 2 accommodation rounds of the obstacle course to familiarize themselves with the condition and the tasks. The physiotherapist could provide physical support if the participants had difficulties with any obstacle. Finally, we instructed the participants to always step over the obstacle and cross the gap with the leg they had spontaneously used in the first round.

According to Redekop et al [31], reliability with an interclass correlation coefficient of 0.90 is given for an average of 6 strides when examining discrete gait parameters in children with CP. Therefore, 8 trials per condition were recorded to have 2 spare measurements if any unexpected errors arose while reviewing the recordings. Once the 8 valid attempts per task were recorded, the participants had a short break, during which they answered the first part of the questionnaire. Subsequently, the same procedure was repeated with the second condition, followed by the second part of the participants’ questionnaire and the proprioception subsection of the FM assessment performed by the investigator. Meanwhile, the physiotherapist completed the therapist’s questionnaire and rated the participant’s FMS and FAQ.

### Data Processing

Vicon data were processed using Nexus Motion Capture Software (version 7.2; Vicon Inc). Processing of the raw data included visual determination and defining gait events like foot strike, foot off, etc. We analyzed the data from the first 6 valid trials for each condition and task. Then, the data were exported to MATLAB R2021a (version 9.10; MathWorks) to calculate the spatiotemporal parameters. For the spatiotemporal parameters, we calculated the mean of the 6 valid trials per task for each participant and condition individually. A negative mean difference between the VR and physical setup indicated a smaller value in the VR setup.

### Outcome Measures

**Figure 3:**
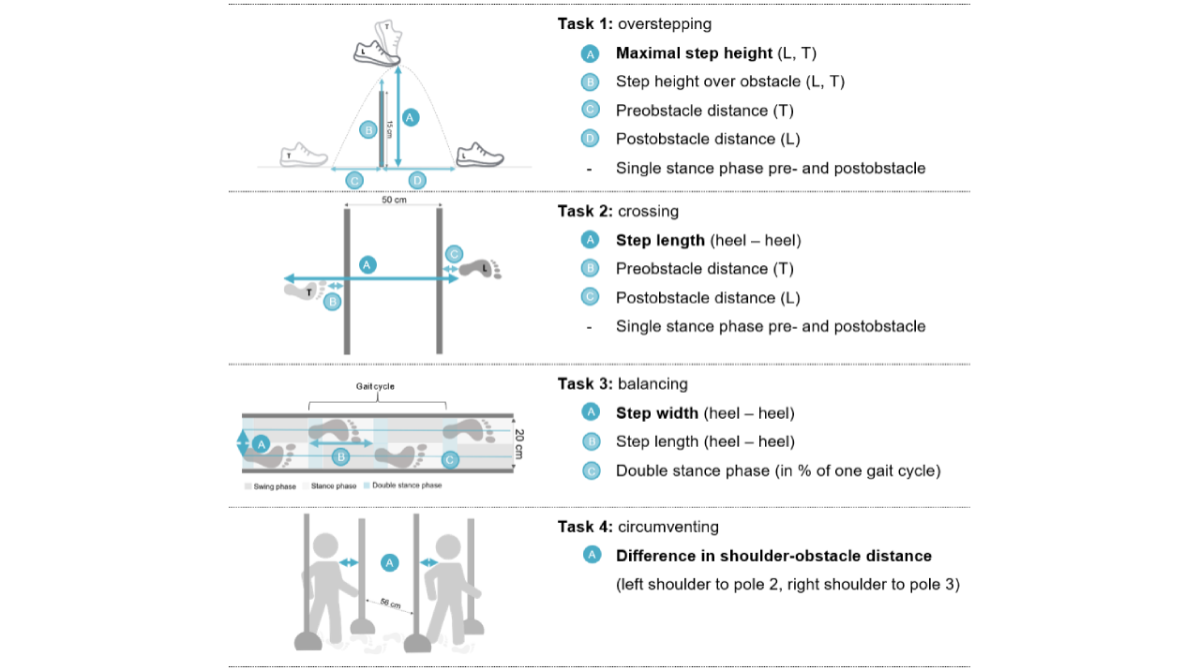
Investigated spatiotemporal parameters for each task. (A) represents the respective primary outcomes. L: leading foot; T: trailing foot.

For the 4 tasks, we selected spatiotemporal parameters (Figure 3) in line with the literature [27,32-35]. We calculated the walking speed, step length and width, and double-stance phase during normal walking with and without the VR HMD. Additionally, we recorded the time to master each task and the number of failures, indicating unsuccessful obstacle negotiations.

The participants answered standardized questions covering their movement ability, spatial presence, and enjoyment during task execution on a visual analog scale (VAS). In addition, the physiotherapists rated the participants’ movement execution, level of engagement, and meaningful use on a 5-point Likert scale.

### Statistical Analysis

Participants’ clinical and functional characteristics are presented using descriptive statistics. A normal distribution could be assumed for the differences between the primary outcomes (Shapiro-Wilk test; *P*>.05). Therefore, the mean differences and their SDs were subsequently calculated. Additionally, the primary spatiotemporal parameters were analyzed using noninferiority testing with 95% CIs and a priori defined margins of noninferiority [36]. The noninferiority margins, which served as boundaries for the 95% CI of the mean differences, were defined for each task by a panel of 15 expert physiotherapists (n=14 women; n=1 men). These margins represent the maximum difference between the VR and the physical setup defined as acceptable while still considering the conditions to be equal [37]. To determine the maximum tolerated deviation, the physiotherapists compared the tasks with everyday life tasks and considered what deviation they would accept in conventional therapy for the respective task. A normal distribution could not be assumed with 15 responses; therefore, we described the margins using nonparametric parameters such as the median and IQR. Descriptive statistics are used to present the participants’ and therapists’ questionnaire responses. Additionally, to analyze the difference in fun between the 2 conditions, we used the Wilcoxon signed rank test with continuity correction.

## Results

### Participants

All patients that were examined for eligibility within the recruitment agreed to participate. In total, 7 girls and 13 boys with different gait disorders participated in this study. Their mean age was 12.0 (SD 3.5) years, and their mean height was 1.46 (SD 0.21) meters. All participants were able to follow the instructions and remained compliant during the measurements. None of the participants reported cybersickness. The spectrum of functional mobility was broad, including FMS levels 3-6 for 5 m and 50 m and 1-6 for 500 m, as well as levels 6-10 of the FAQ. However, most participants could walk independently on all surfaces without any walking device, for at least short to medium distances (FMS 5 m and FMS 50 m ≥5 each). Participants’ lower extremity proprioception (FM score) ranged from normal to mildly impaired. A total of 9 of the 20 participants had already used a VR HMD at least once before this study. Participants’ clinical and functional characteristics are presented in Table 1.

**Table 1:** Clinical and functional characteristics of the participants.

ID	Sex	Age (years)	Height (cm)	Diagnosis^a^	FMS^b^	FAQ^c^	FM^d^	Mobility aid^e^
1	Male	13.4	155	Unilateral spastic cerebral palsy (I)	6/5/5	9	93%^f^	None
2	Male	13.4	157	Unilateral spastic cerebral palsy (I)	6/6/6	9	100%^f^	None
3	Female	17.0	165	Vasomotor dysregulation with neurological involvement	5/3/3	9	93%^f^	Forearm crutches
4	Male	9.3	135	Brain tumor	6/6/6	9	94%	None^g^
5	Male	14.2	166	Polytrauma	6/6/5	9	94%	None
6	Male	17.8	176	Spinal tumor with neurological involvement	6/5/5	8	94%	None^g^
7	Male	8.0	141	Stroke	6/6/6	10	100%	None
8	Female	16.8	163	Myasthenia gravis	6/6/5	9	94%	None
9	Female	8.0	121	Rhabdomyolysis	6/6/5	9	94%	None
10	Male	6.6	110	Brain tumor	6/6/6	9	88%	None
11	Male	13.6	148	Myelomeningocele	5/3/1	7	100% ^f^	Forearm crutches
12	Female	10.9	147	Stroke	6/6/6	9	100%	None
13	Male	15.1	160	Myelomeningocele	3/3/1	6	100%	Forearm crutches
14	Male	14.5	165	Stroke	6/6/6	9	100%	None
15	Female	13.4	171	Ataxia	6/6/5	9	100%	None
16	Male	11.6	145	Bilateral spastic cerebral palsy (I)	6/6/5	9	100%	None
17	Male	7.0	112	Arthrogryposis Multiplex Congenita	5/5/2	7	94%	None^g^
18	Male	9.7	118	Myelomeningocele	5/5/1	9	88%	None
19	Female	8.3	121	Unilateral spastic cerebral palsy (I)	6/6/6	10	100%	None
20	Female	10.9	142	Brain tumor	6/5/5	8	94%	None^g^

^a^In children and adolescents diagnosed with cerebral palsy, the Gross Motor Function Classification System Level is given in parentheses.

^b^FMS: Functional Mobility Scale 5/50/500 m.

^c^FAQ: Gillette Functional Assessment Questionnaire-walking scale.

^d^FM: Fugel-Meyer assessment.

^e^Mobility aid used in both conditions.

^f^Due to restricted movements in certain joints or due to pain, not all movements of the FM could be performed by these participants. Therefore, for these participants, the relative value is not calculated from the maximum score (16 points), but from the individual maximum score (8-14 points).

^g^Did not need a mobility aid, but needed close supervision of their physiotherapist.

The participants had to walk the obstacle course from 8 to 16 times to obtain 6 valid trials per task. This resulted in 25-39 recordings per participant for the entire measurement. The most frequent reason why a trial was considered invalid was crossing the obstacle with the wrong leading foot. Furthermore, some attempts were declared invalid when the instructions were not followed or the recording of the markers failed. There were no missing data, except for participant 9 (only 5 valid crossing task trials in the physical setup) and participant 10 (only 5 valid overstepping task trials in the physical setup).

### Spatiotemporal Parameters

**Figure 4:**
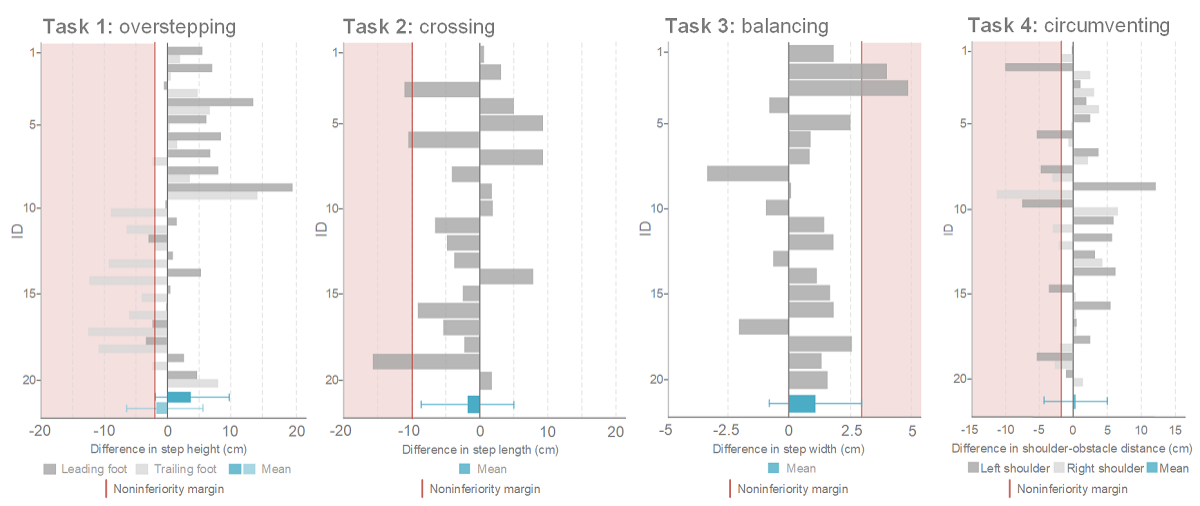
Differences per participant and task for the primary outcomes. The blue bars represent the mean differences and SD over all participants.

The differences between the VR and the physical condition varied widely between the participants and tasks (Figure 4).

During normal walking, step length and gait speed decreased, and step width slightly increased in the VR condition compared to the physical setup (Table 2). In task 1, participants lifted the leading foot 3.77 cm higher and the trailing foot 1.75 cm lower in the VR setup when overstepping the obstacle. In task 2, they decreased the step length by 1.75 cm in the VR setup. As in normal walking, step width and the double stance phase increased, while step length decreased in the VR setup of the balancing task. For task 4, the distance from the shoulder to the obstacle did not differ between the 2 conditions.

**Table 2:** Spatiotemporal parameters for the conditions and tasks.

Task and parameter		Physical setup	Virtual reality setup	Difference^a^	
**Task 0: normal walking, mean (SD)**					
	Step length (cm)	60.44 (10.22)	54.91 (7.11)	–5.53 (7.14)	
	Step width (cm)	9.29 (3.92)	9.48 (3.07)	0.19 (2.07)	
	Gait speed (m/second)	1.10 (0.23)	0.95 (0.20)	–0.15 (0.24)	
	Double stance phase (%)	24.75 (4.41)	27.48 (3.82)	2.72 (4.21)	
**Task 1: overstepping**					
	Max step height (L^b^; cm)^c^, mean (SD)	27.53 (4.74)	31.31 (7.21)	3.77 (5.69)	
	Max step height (T^d^; cm)^c^, mean (SD)	28.30 (6.27)	26.55 (8.47)	–1.75 (7.07)	
	Step height over obstacle (L; cm), mean (SD)	24.77 (5.18)	25.30 (8.29)	0.53 (5.64)	
	Step height over obstacle (T; cm), mean (SD)	25.32 (5.61)	18.80 (9.06)	–6.52 (8.28)	
	Preobstacle distance (T; cm), mean (SD)	16.45 (7.66)	10.17 (9.01)	–6.28 (5.60)	
	Postobstacle distance (L; cm), mean (SD)	19.60 (5.67)	24.45 (6.97)	4.85 (5.58)	
	Single stance preobstacle (T; seconds), mean (SD)	0.70 (0.17)	0.75 (0.16)	0.05 (0.10)	
	Single stance postobstacle (L; seconds), mean (SD)	0.62 (0.14)	0.60 (0.10)	–0.01 (0.12)	
	Total time (seconds), mean (SD)	3.64 (1.49)	4.03 (1.16)	0.39 (0.84)	
	Total failures max step height <16 cm (L), n (number of children)	1 (1)^e^	4 (2)^e^	3 (1)^e^	
	Total failures max step height <16 cm (T), n (number of children)	1 (1)^e^	15 (3)^e^	14 (2)^e^	
**Task 2: crossing**					
	Step length (cm)^c^, mean (SD)	83.81 (7.11)	82.06 (9.32)	–1.75 (7.22)	
	Preobstacle distance (T; cm), mean (SD)	6.36 (4.55)	–5.91 (8.27)	–12.27 (8.87)	
	Postobstacle distance (L; cm), mean (SD)	3.29 (5.99)	13.82 (7.50)	10.53 (6.96)	
	Single stance preobstacle (T; seconds), mean (SD)	0.61 (0.14)	0.69 (0.17)	0.08 (0.15)	
	Single stance postobstacle (L; seconds), mean (SD)	0.54 (0.08)	0.54 (0.09)	0.00 (0.07)	
	Total time (seconds), mean (SD)	4.05 (1.26)	4.69 (1.11)	0.64 (0.79)	
	Total failures step length <51 cm, n (number of children)	14 (7)^e^	31 (10)^e^	17 (3)^e^	
**Task 3: balancing**					
	Step width (cm)^c^, mean (SD)	5.36 (2.92)	6.41 (2.69)	1.05 (1.93)	
	Step length (cm), mean (SD)	52.73 (8.51)	47.31 (11.56)	–5.41 (8.45)	
	Double stance phase (%), mean (SD)	28.58 (5.35)	32.55 (6.05)	3.97 (6.39)	
	Total time (seconds), mean (SD)	4.44 (1.43)	5.31 (1.72)	0.87 (1.88)	
	Total failures step width >19 cm, n (number of children)	6 (3)^e^	5 (3)^e^	–1 (0)^e^	
**Task 4: circumventing**					
	Minimal shoulder-obstacle distance (cm)^c^, mean (SD)	10.66 (3.36)	10.41 (3.77)	0.25 (4.44)	
	Total time (seconds), mean (SD)	5.25 (2.48)	5.76 (1.98)	0.50 (1.51)	
	Total failures minimal distance <2 cm, n (number of children)	3 (3)^e^	13 (7)^e^	10 (4)^e^	

^a^The differences were calculated by subtracting the value of the physical setup from the value of the virtual reality setup. Consequently, negative differences indicate a lower value for the virtual reality setup.

^b^L: leading foot.

^c^Primary outcomes (also used to define the number of fails).

^d^T: trailing foot.

^e^The number of children that made these fails.

### Noninferiority Analysis

**Figure 5:**
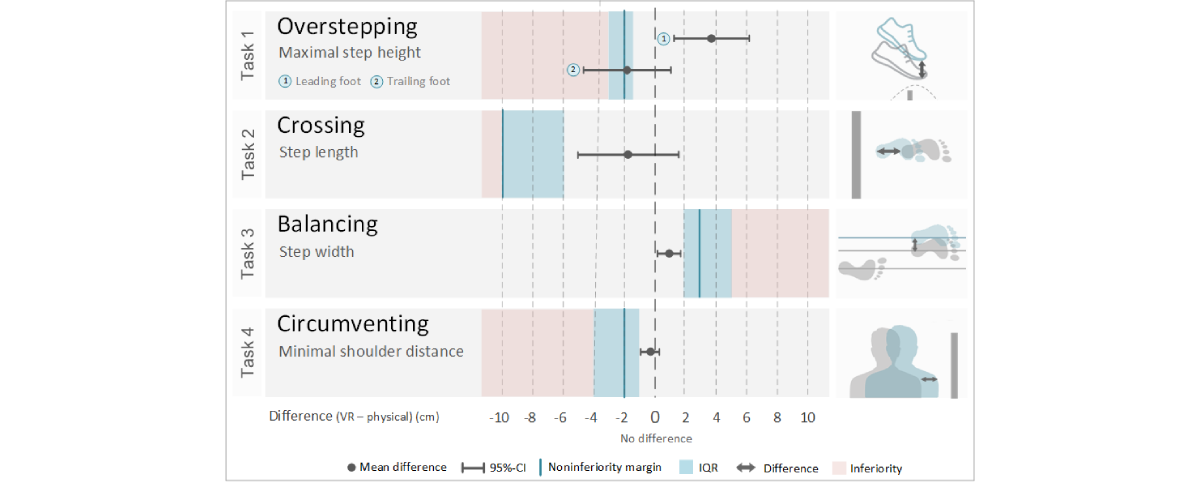
Noninferiority analysis for the primary outcomes. A negative mean difference indicates a smaller value in the virtual reality (VR) setup than in the physical setup. The noninferiority margins in blue represent the maximum difference between the two conditions while still considering the conditions to be equal. As long as the 95% CI of the mean difference does not exceed this margin, the VR setup is noninferior to the physical setup. Inferiority of the VR setup is assumed when the 95% CI touches the red inferiority area and, at the same time, does not cross the line of no difference between the two conditions.

We applied noninferiority analyses [37] to compare the differences in the primary outcomes between the VR and physical setups for each task according to the a priori defined noninferiority margins. As depicted in Figure 5, the noninferiority analysis revealed noninferiority for the leading foot and was inconclusive for the trailing foot when overstepping the obstacle. For crossing, balancing, and circumventing, the results of the statistical analysis showed noninferiority in all cases.

### Questionnaires

**Figure 6:**
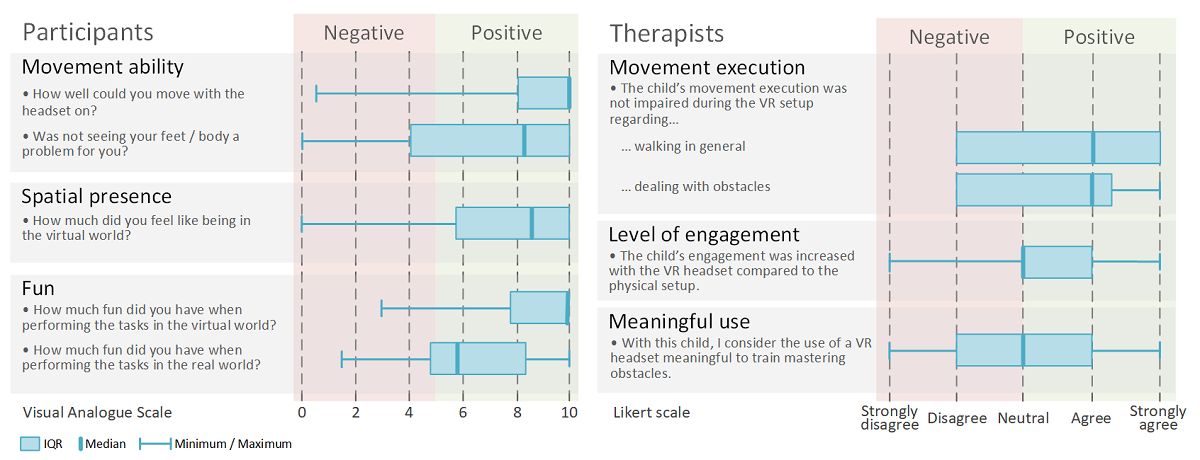
The participants’ and therapists’ views on the use of the virtual reality (VR) head mounted display (HMD) in physiotherapy.

When asked how well the participants could move around using the HMD, 75% (15/20) of the participants scored ≥8 and did not feel restricted in their ability to move around. Not being able to see their body or feet was no problem (score ≥7) for most (14/20, 70%; Figure 6) participants. Most participants (14/20, 70%) felt physically present in the virtual scenario (score ≥7), even if the environment and the objects did not seem entirely realistic to them. The participants had fun in both conditions; however, the VR setup was rated significantly better (*P<*.001).

According to the therapists, movement execution during the VR setup was not impaired in 65% (13/20) of the participants when walking normally or dealing with obstacles. The most common reason why therapists considered mild impairment in movement execution while wearing the HMD was a more cautious and slower gait pattern. The therapists perceived the level of engagement in the VR setup to be lower in 4 participants, similar in 7 participants, and higher in 9 participants. Therapists had ambivalent views regarding the meaningfulness of using VR to train for mastering obstacles. Reasons for considering the application meaningful included increased enthusiasm for movement, the challenge of altered visual control, and, therefore, the increased awareness of the children’s bodies. Reduced speed, reduced focus on the given instructions, lack of feeling the edges of the obstacles, and consequences, such as stumbling when not lifting the foot high enough, were reasons against VR being a meaningful application for some participants. The balancing task was the task most often considered meaningful by the therapists.

## Discussion

### Principal Findings

This study aimed to provide information on whether a VR setup is feasible and motivating to induce and practice movements that are needed to master real obstacles in children and adolescents with gait disorders. Furthermore, this study aimed to evaluate which kinds of everyday walking activities are appropriate to be practiced in such a VR setup. To achieve these goals, a virtual and a physical condition, the latter resembling therapeutic setup, were compared with each other. We chose a noninferiority analysis to quantify the differences between spatiotemporal parameters defined a priori. This noninferiority analysis revealed that for 3 of the 4 walking tasks encountered in everyday life, mastering the virtual obstacles provided by an HMD was noninferior to mastering the physical obstacles. Thus, the results suggest that children and adolescents with gait disorders can practice crossing a wide gap, balancing on a narrow area, and circumventing stationary obstacles appropriately in a virtual setup. Furthermore, overstepping a virtual obstacle with the leading foot was also noninferior; only the results for the trailing foot were inconclusive.

### Comparison to Previous Work

#### Normal Walking

The participants walked slower in the VR compared to the physical setup, which corresponds to the findings of Almajid et al [38] and Horsak et al [39]. Almajid et al [38] found that younger and older adults needed significantly more time to perform the timed up-and-go test when wearing an HMD, even without the projection of a virtual scene. In the study of Horsak et al [39], healthy individuals also demonstrated a slower walking pattern when walking in an overground VR environment compared to a real environment. This effect should be considered when wearing an HMD in rehabilitation. Still, the mean gait speed during normal walking in the VR setup was within the range of self-selected walking speed in children aged between 7 and 14 years with CP (GMFCS levels I and II) and TBI [25-27]. Although the participants’ FMS and FAQ values were in the upper range of the scales, their gait speed in the physical setup was still below the average of typically developing youths [40]. The reduced gait speed in the VR setup was accompanied by a decreased step length and a slightly prolonged double stance phase.

The mean step length in both conditions was above the average step length of 50 cm reported for children aged between 7 and 14 years with CP (GMFCS stages I and II) and TBI, but below the average step length of 68 cm reported for typically developing children of the same age [25-27]. The double-stance phase of our participants during normal walking was in both conditions remarkably longer than in typically developing youths aged between 5 and 21 years [40]. Several therapists observed that the movements of their patients were constrained at the beginning of wearing the HMD, especially during normal walking. This could be because more than half of the participants had never worn a VR HMD before participating in this study. However, the difference in double stance time between the VR and physical setup was considerably smaller compared to the difference in double stance time between typically developing individuals and individuals with blindness [41].

#### Overstepping

First, the noninferiority analysis demonstrated that the maximal step height of the leading foot when stepping over the virtual obstacle was noninferior compared to the physical obstacle. This indicated that participants raised their leading foot to the same height when overstepping the virtual obstacle. However, they lifted their trailing foot considerably less high in the VR condition. This finding is supported by a study by Hagio and Kouzaki [42], in which healthy adults overstepped a virtual and physical obstacle. While the vertical height of the leading foot correlated highly (*r*=0.77) between the VR and physical condition, the correlation was lower for the trailing foot (*r*=0.47). As Kim et al [43] describe, an explanation for the difference between the leading and trailing foot in the VR setup could be the missing visual information regarding the height of the foot and, therefore, not being able to correct its height. Further results from Hagio and Kouzaki [42] suggest that visuomotor transformation in the leading leg contributes to a motor plan for trailing limb toe trajectories while stepping over an obstacle.

#### Crossing

Second, although the primary outcome parameters were mostly comparable between the virtual and physical setups, the movement was slightly displaced when overstepping or crossing the obstacles in the VR setup. Participants stepped too close to the obstacle or even over the edge of the obstacle. In general, however, the steps were almost the same length and height in the VR and physical setups, just at different locations. As the HMD blocks out the physical world, a lack of spatial information about the environment and the body’s state relative to the environment could be a reason for the slightly displaced movement execution in the VR condition. However, most participants indicated on the VAS that not seeing their feet or body was not a problem for them. Furthermore, almost half of the participants scored 100% on the FM assessment, which tests the proprioception of the lower extremities. Nevertheless, using a fully immersive VR, Kim et al [43] investigated how visual information about the lower extremities is integrated with information about the environment to facilitate successful obstacle avoidance in healthy young adults. Their study revealed that visual information about the lower extremities promoted more consistent behavior while stepping over an obstacle.

#### Balancing

Third, in both conditions of the balancing task, the step length was slightly decreased, and the double stance phase increased compared to the corresponding normal walking condition. As reduced step length and prolonged double stance phase are considered indicators of reduced balance [41], we can assume that the participants made a real effort to balance over the physical and virtual obstacles. Although the participants rated this task as rather difficult, they produced only a small number of failures. The step width, which we considered crucial for successfully completing the balance task, was, on average, 1 cm larger in the VR than in the physical setup. However, the noninferiority analysis illustrated that the step width in the VR setup was noninferior to that of the physical condition. Therefore, we assume that the participants successfully balanced over the obstacle in VR and in reality.

#### Circumventing

Fourth, when moving in public areas, it becomes essential to circumvent stable objects or moving people, have a stable base of support, and balance in a narrow space. Several studies have investigated the critical point (the ratio between aperture width and shoulder width at which a shoulder rotation occurs at the time of crossing) and safety margin (the space that is maintained between the shoulders and the obstacles at the time of crossing) for aperture crossing [30,44]. Whenever the participants had to rotate their shoulders, they maintained a larger safety margin when crossing [44]. For example, the critical point for circumventing poles, calculated from the mean shoulder width of the participants and the distance between the poles, was a ratio of 1.3 [44]. The present study’s ratio between the aperture width and the mean shoulder width equaled 1.6. Assuming that participants did not rotate their shoulders at such a ratio, the safety margin was slightly less than the 30 cm observed in the study of Hackney et al [30]. However, the safety margins of 10 cm of the VR and physical setup equal those of young, healthy adults who had to avoid poles with an aperture/shoulder width ratio of 1.3 [44]. The results of the noninferiority analysis suggest that participants successfully circumvented the obstacles in the VR setup. In addition, Hackney et al [45] recently showed that individuals who had to avoid obstacles in a virtual scenario wearing an HMD behave similarly with virtual poles and avatars, indicating generalization to a wide range of applications in VR.

#### Questionnaires

In summary, the participants were very positive toward training walking tasks in a VR setting. Due to its game-like features, the participants experienced significantly more fun in the VR than in the physical setup. How VR-assisted physical therapy might affect a participant's enjoyment and motivation over time needs to be investigated in the future. The physiotherapists did not observe a difference in the participants’ engagement level between the VR and physical setup, indicating that the participants made similar efforts in both conditions. Thus, a comparison between the 2 conditions was feasible.

### Limitations

This study has several limitations. First, the group size of 20 participants was rather small. However, it is in line with recommendations [22], as the purpose of this study was to provide information on whether a VR setup is feasible and motivating to induce movements that are needed to master real obstacles and which kinds of everyday walking activities are appropriate to be practiced in such a VR setup. To examine the appropriateness and effectiveness of VR training, more participants would have to be included in the next study. Despite the considerable heterogeneity of this study, noninferiority could still be shown in 3 tasks.

Second, even though the dimensions and locations of the obstacles did match in both conditions, the different visualizations of the physical setup and the VR setup could have impacted the participants’ gait. However, this limitation was chosen intentionally, as we wanted the obstacles to look like they would appear in future applications.

Third, a panel of experts decided on specific margins to define noninferiority, as no reliable reference values for the noninferiority analyses existed in the literature. In order to minimize this limitation for a further project, additional external experts could be asked and added to the panel.

Fourth, the gait laboratory is frequently used for clinical gait analysis. Therefore, the Vicon cameras pointed to the middle of the room. Since the recording area for this study was slightly broader, some markers disappeared at times from the measurement volume, which is one reason why some participants had to complete more than 8 rounds to record sufficient valid trials. Consequently, the high number of repetitions might have bored and fatigued some participants, which might have decreased their concentration toward the end. With verbal input for the participants and breaks between the trials if needed, we tried to keep the number of trials and the fatigue of the participants as low as possible.

Fifth, a slight misalignment between the real and virtual setups might have introduced an unknown error in calculating the parameters. We calibrated the alignment immediately before putting the HMD on the participant’s head to minimize this error.

Sixth, the feet were not visible to the participants in the VR condition. We assume that a lack of spatial information rather than impairments in proprioception might have caused failures such as stepping over the edge, as the FM assessment did not indicate major lower limb proprioception impairments in the participants. A further study investigating the influence of foot projection in VR could provide further information regarding the influence of the visability of the feet.

### Conclusions

This is the first study showing that children and adolescents with gait disorders master various obstacle tasks, such as overstepping a bar, crossing a wide gap, balancing on a narrow area, and circumventing stationary obstacles, similarly in VR and physical conditions. Only the results for the trailing foot in the overstepping task were inconclusive. Therefore, we conclude that using a VR setup to practice mastering obstacles with children and adolescents with gait disorders is feasible and motivates them to practice everyday walking tasks. In the long run, the feasibility of using HMDs in a clinical therapy setting, patient motivation over a longer period of time, the appropriateness and effectiveness of such VR interventions, and identifying potential responders to such interventions require further investigations.

## Abbreviations

CP: cerebral palsy
FAQ: Functional Assessment Questionnaire
FM: Fugl-Meyer
FMS: Functional Mobility Scale
GMFCS: Gross Motor Function Classification System
HMD: head-mounted display
TBI: traumatic brain injury
VAS: visual analog scale
VR: virtual reality

## Data Availability

The data sets generated during and/or analyzed during this study are available from the corresponding author on reasonable request.

## References

[1] Katz-Leurer M, Rotem H, Keren O, Meyer S (2009). Balance abilities and gait characteristics in post-traumatic brain injury, cerebral palsy and typically developed children. Dev Neurorehabil.

[2] Frisch D, Msall ME (2013). Health, functioning, and participation of adolescents and adults with cerebral palsy: a review of outcomes research. Dev Disabil Res Rev.

[3] Rast FM, Labruyère R (2020). ICF mobility and self-care goals of children in inpatient rehabilitation. Dev Med Child Neurol.

[4] Rosenbaum P, Gorter JW (2012). The 'F-words' in childhood disability: I swear this is how we should think!. Child Care Health Dev.

[5] Cipresso P, Giglioli IAC, Raya MA, Riva G (2018). The past, present, and future of virtual and augmented reality research: a network and cluster analysis of the literature. Front Psychol.

[6] Bailey JO, Bailenson JN (2017). Chapter 9—Immersive virtual reality and the developing child. Cognitive Development in Digital Contexts.

[7] Bainbridge WS (2007). The scientific research potential of virtual worlds. Science.

[8] Perez-Marcos D (2018). Virtual reality experiences, embodiment, videogames and their dimensions in neurorehabilitation. J Neuroeng Rehabil.

[9] Levac D, Rivard L, Missiuna C (2012). Defining the active ingredients of interactive computer play interventions for children with neuromotor impairments: a scoping review. Res Dev Disabil.

[10] Granic I, Lobel A, Engels RCME (2014). The benefits of playing video games. Am Psychol.

[11] Sharar SR, Carrougher GJ, Nakamura D, Hoffman HG, Blough DK, Patterson DR (2007). Factors influencing the efficacy of virtual reality distraction analgesia during postburn physical therapy: preliminary results from 3 ongoing studies. Arch Phys Med Rehabil.

[12] Morrongiello BA, Corbett M, Beer J, Koutsoulianos S (2018). A pilot randomized controlled trial testing the effectiveness of a pedestrian training program that teaches children where and how to cross the street safely. J Pediatr Psychol.

[13] Adamo-Villani N, Wilbur R, Wasburn M (2008). Gender differences in usability and enjoyment of VR educational games: a study of SMILE™.

[14] Newbutt N, Bradley R, Conley I (2020). Using virtual reality head-mounted displays in schools with autistic children: views, experiences, and future directions. Cyberpsychol Behav Soc Netw.

[15] Parsons S (2015). Learning to work together: designing a multi-user virtual reality game for social collaboration and perspective-taking for children with autism. Int J Child Comput Interact.

[16] Shema-Shiratzky S, Brozgol M, Cornejo-Thumm P, Geva-Dayan K, Rotstein M, Leitner Y, Hausdorff JM, Mirelman A (2019). Virtual reality training to enhance behavior and cognitive function among children with attention-deficit/hyperactivity disorder: brief report. Dev Neurorehabil.

[17] Mon-Williams M (2017). Is virtual reality bad for our health? The risks and opportunities of a technology revolution. Medium.

[18] Tychsen L, Foeller P (2020). Effects of immersive virtual reality headset viewing on young children: visuomotor function, postural stability, and motion sickness. Am J Ophthalmol.

[19] Ravi DK, Kumar N, Singhi P (2017). Effectiveness of virtual reality rehabilitation for children and adolescents with cerebral palsy: an updated evidence-based systematic review. Physiotherapy.

[20] Chen Y, Fanchiang HD, Howard A (2018). Effectiveness of virtual reality in children with cerebral palsy: a systematic review and meta-analysis of randomized controlled trials. Phys Ther.

[21] Ammann-Reiffer C, Kläy A, Keller U (2022). Virtual reality as a therapy tool for walking activities in pediatric neurorehabilitation: usability and user experience evaluation. JMIR Serious Games.

[22] Macefield R (2009). How to specify the participants groups size for usability studies: a practitioner's guide. J Usability Stud.

[23] Ammann-Reiffer C, Bastiaenen CHG, Klöti C, van Hedel HJA (2019). Concurrent validity of two gait performance measures in children with neuromotor disorders. Phys Occup Ther Pediatr.

[24] Fugl-Meyer AR, Jääskö L, Leyman I, Olsson S, Steglind S (1975). The post-stroke hemiplegic patient. 1. a method for evaluation of physical performance. Scand J Rehabil Med.

[25] Fosdahl MA, Jahnsen R, Kvalheim K, Holm I (2019). Medicina (Kaunas).

[26] Katz-Leurer M, Rotem H, Keren O, Meyer S (2009). The relationship between step variability, muscle strength and functional walking performance in children with post-traumatic brain injury. Gait Posture.

[27] Kimoto M, Okada K, Sakamoto H, Kondou T (2017). The association between the maximum step length test and the walking efficiency in children with cerebral palsy. J Phys Ther Sci.

[28] Weiten J (2004). Auxiologische Untersuchung bei Kindern mit Wachstumshormonmangel vor und unter der Substitutionstherapie mit Wachstumshormon. Humboldt-Universität zu Berlin. [Doctoral thesis].

[29] Spranger J, Ochsenfarth A, Kock HP, Henke J (1968). Anthropometrische Normdaten im Kindesalter [Anthropometric standards in childhood]. Z Kinderheilkd.

[30] Hackney AL, Vallis LA, Cinelli ME (2013). Action strategies of individuals during aperture crossing in nonconfined space. Q J Exp Psychol (Hove).

[31] Redekop S, Andrysek J, Wright V (2008). Single-session reliability of discrete gait parameters in ambulatory children with cerebral palsy based on GMFCS level. Gait Posture.

[32] Creem-Regehr SH, Gill DM, Pointon GD, Bodenheimer B, Stefanucci JK (2019). Mind the gap: gap affordance judgments of children, teens, and adults in an immersive virtual environment. Front Robot AI.

[33] Cappellini G, Sylos-Labini F, MacLellan MJ, Assenza C, Libernini L, Morelli D, Lacquaniti F, Ivanenko Y (2020). Locomotor patterns during obstacle avoidance in children with cerebral palsy. J Neurophysiol.

[34] Berard JR, Vallis LA (2006). Characteristics of single and double obstacle avoidance strategies: a comparison between adults and children. Exp Brain Res.

[35] Chang MC, Lee BJ, Joo NY, Park D (2021). The parameters of gait analysis related to ambulatory and balance functions in hemiplegic stroke patients: a gait analysis study. BMC Neurol.

[36] Schumi J, Wittes JT (2011). Through the looking glass: understanding non-inferiority. Trials.

[37] Stefanos R, Graziella DA, Giovanni T (2020). Methodological aspects of superiority, equivalence, and non-inferiority trials. Intern Emerg Med.

[38] Almajid R, Tucker C, Keshner E, Vasudevan E, Wright WG (2021). Effects of wearing a head-mounted display during a standard clinical test of dynamic balance. Gait Posture.

[39] Horsak B, Simonlehner M, Schöffer L, Dumphart B, Jalaeefar A, Husinsky M (2021). Overground walking in a fully immersive virtual reality: a comprehensive study on the effects on full-body walking biomechanics. Front Bioeng Biotechnol.

[40] Voss S, Joyce J, Biskis A, Parulekar M, Armijo N, Zampieri C, Tracy R, Palmer AS, Fefferman M, Ouyang B, Liu Y, Berry-Kravis E, O'Keefe JA (2020). Normative database of spatiotemporal gait parameters using inertial sensors in typically developing children and young adults. Gait Posture.

[41] Hallemans A, Ortibus E, Truijen S, Meire F (2011). Development of independent locomotion in children with a severe visual impairment. Res Dev Disabil.

[42] Hagio S, Kouzaki M (2020). Visuomotor transformation for the lead leg affects trail leg trajectories during visually guided crossing over a virtual obstacle in humans. Front Neurosci.

[43] Kim A, Kretch KS, Zhou Z, Finley JM (2018). The quality of visual information about the lower extremities influences visuomotor coordination during virtual obstacle negotiation. J Neurophysiol.

[44] Hackney AL, Cinelli ME, Frank JS (2015). Does the passability of apertures change when walking through human versus pole obstacles?. Acta Psychol (Amst).

[45] Hackney AL, Cinelli ME, Warren WH, Frank JS (2020). Are avatars treated like human obstacles during aperture crossing in virtual environments?. Gait Posture.

